# Corn Cob Ash versus Sunflower Stalk Ash, Two Sustainable Raw Materials in an Analysis of Their Effects on the Concrete Properties

**DOI:** 10.3390/ma15030868

**Published:** 2022-01-24

**Authors:** Adrian Alexandru Șerbănoiu, Cătălina Mihaela Grădinaru, Radu Muntean, Nicanor Cimpoeșu, Bogdan Vasile Șerbănoiu

**Affiliations:** 1 Faculty of Civil Engineering and Building Services, “Gheorghe Asachi” Technical University of Iași, 700050 Iași, Romania; serbanoiu.adrian@tuiasi.ro; 2 Faculty of Civil Engineering, Transilvania University of Brașov, 500152 Brașov, Romania; radu.m@unitbv.ro; 3 Faculty of Material Science and Engineering, “Gheorghe Asachi” Technical University of Iași, 700050 Iași, Romania; nicanor.cimpoesu@academic.tuiasi.ro; 4 Faculty of Architecture “G.M. Cantacuzino”, “Gheorghe Asachi” Technical University of Iași, 700050 Iași, Romania; bogdan-vasile.serbanoiu@academic.tuiasi.ro

**Keywords:** agro-waste, eco-friendly material, sustainable building materials, ecological concrete

## Abstract

The increased CO_2_ emissions determined by the cement industry led to continuous and intensive research on the discovery of sustainable raw materials with cementitious properties. One such raw material category is agricultural waste. This study involved research on the effects of corn cob ash and sunflower stalk ash, respectively, on compressive strength measured after 28 days and 3 months, the flexural and splitting tensile strengths, the resistance to repeated freeze–thaw cycles, and on the resistance to chemical attack of hydrochloric acid of the concrete. A 2.5% and 5% replacement of the cement volume with corn cob ash (CCA) of A and B quality was applied, and with sunflower stalk ash (SSA) at A and B quality, respectively. The obtained results revealed that CCA and SSA decreased the compressive and tensile strength, but led to higher resistance of the concrete on repeated freeze–thaw cycles and to hydrochloric acid. The mixes with 2.5% SSA at A quality obtained the best results regarding splitting the tensile strength and resistance to repeated freeze–thaw cycles, the mixes with 2.5% SSA at B quality led to the highest resistance to hydrochloric acid, and those with 2.5% CCA at A quality led to the best values of compressive strength and flexural tensile strength.

## 1. Introduction

The world’s cement production has been estimated to around 4.1 billion metric tons [1]; it emitted around 2.3 gigatons of CO_2_ in 2019, this value being around 7% of total CO_2_ emissions [2], and it is expected to reach 4.83 billion metric tons in 2030 [3]. A solution for decreasing the CO_2_ emissions determined by the cement industry is its replacement by supplementary cementitious materials (SCMs). These SCMs can contribute to reduce the environmental impact and costs related to the cement and concrete industry, since they need less process heating and emit smaller CO_2_ levels [4], enhance sustainability, and improve some of the concrete’s properties [5]. The SCMs category includes, among many others, the vegetal ashes obtained from the burning of different plants. A few examples of such vegetal ashes that were studied as SCMs in concrete composition are: rice husk ash [6,7,8], sugarcane bagasse ash [9,10], corn cobs [5,11,12], wheat [13,14], and many others. Previous studies showed that lignocellulosic ash containing significant rates of silica and alumina can develop pozzolanic activity in the presence of calcium hydroxide, leading to improved properties of concrete [15,16]. Maize and sunflower, important crops that have an annual global cultivated area of around 178 × 10^6^ ha [17], and 26.03 × 10^6^ ha [18], respectively, represent significant and widely spread sources of lignocellulosic materials. Maize crops are cultivated mainly in China, USA, South Africa, and Eastern Europe, whereas sunflower crops are grown in southern South America, Southern Europe, South Africa, and South and European Russia [19].

Corn cob ash is a SCM studied quite intensively lately, as it has proven pozzolanic properties in various studies that have been conducted on the use of corn cobs as a cement partial replacement in concrete, but the results have been mixed. Adesanya, 1996 [20], and Binici et al., 2008 [21], showed that corn cob ash (CCA) determined less water absorption and improved resistance to sulfate attack. The studies led by Adesanya, 1996 [20], Adesanya and Raheem, 2009 [12], Kamau et al., 2017 [22], and Memon and Khan, 2018 [23], concluded that CCA determined a significant decrease in concrete compressive strength. Adesanya and Raheem, 2009 [12], found that CCA decreases the concrete slump, whereas Kamau et al., 2016 [5], found that it increases it. In 2012, Olafusi and Olutoge [24] studied the strength characteristics of corn cob ash concrete and concluded that the concrete did not reach its design strength after 28 days, and this depends on the pozzolanic activity of the corn cob ash. From the results of the various tests carried out by Mujedu et al., 2014 [25], it can be concluded that the combination of corn cob ash (CCA) and sawdust ash (SDA) is a suitable material for pozzolana, because it has a combination of more than 70% (SiO_2_ + Al_2_O_3_ + Fe_2_O_3_) to meet the requirements of this material. The compressive strength of concrete increases with curing time and decreases as the percentage combination of CCA and SDA increases up to 10% of ordinary Portland cement in concrete to obtain the greatest strength gain. Although the strength of CCA-SDA concrete is lower than that used as reference, it can still be used for general concrete projects where strength is not important, such as floors, mortars, and mass concrete [25]. According to Ahangba and Tiza, 2016 [26], when 10% CCA is used instead of cement, the cement setting time increases from 258 min to 277 min). Substitution beyond this range will reduce the strength of concrete and cannot be controlled. This kind of substitution can also be used in building walls and beam units to reduce the use of cement and its high cost. Analyzing the research of Owolabi et al., 2015 [27], it can be pointed out that the workability of fresh corn cob ash concrete decreases with the increases in corn cob ash content, its compressive strength of concrete decreases with increasing CCA replacement rate, but increases with increasing age of cure, and to obtain the best compressive strength of the concrete, it is recommended to replace 5% of the cement with corn cob ash. The studies developed on CCA concrete focused mainly on the mechanical properties of fresh and hardened material, and on the resistance to sulphate attack. Fewer studies were conducted on the hydration process and chloride resistance of this type of concrete. Shakouri et al., 2020 [28], studied the effects of CCA as a cement replacement between 3% and 20% in concrete, and they found that it negatively affects the cement hydration and decreases the compressive strength and the chloride ion permeability of the concrete. The durability of CCA concrete to chloride corrosion represents an important problem that can reduce infrastructure service life worldwide [28].

Sunflower stalk ash (SSA) is a SCM much less studied than CCA, to our knowledge. Aksoğan et al. (2016) [14] studied the compressive strength, abrasive resistance, and linear absorption coefficient of concrete made with barite, colemanite, and SSA as a cement replacement and they found that the optimum replacement rate was 2.5% of SSA. A higher rate of 5% of SSA increased the concrete resistance to 5% sodium sulfate solution during an 180 days test. They also found that SSA improved the concrete behavior during the freeze–thaw process [14]. Darweesh (2020) [29] studied the physical, chemical, and mechanical properties of cement pastes with SSA, and found that SSA increases the compressive strength, the CSH amount, and decreases the free lime content, and that 24 wt% was the optimum rate of cement replacement with SSA, with higher rates as 30 wt% having negative effects on the cement paste properties.

The aim of this study was to study the effects of corn cob ash and sunflower stalk ash obtained by two methods on the mechanical and some durability properties of the normal concrete. The study novelty consists of the comparative analysis of these two types of vegetal ashes, since to our knowledge very few studies were carried out on concrete with sunflower stalks, and we did not find any with which to compare directly their performances. For obtaining the CCA and SSA, the same methods were applied in order to have a clear view of the differences given by the two plants. The research implied the development of eight mixes with 2.5 vol.% and 5 vol.% cement replacement with CCA at A and B quality, and with SSA at A and B quality. The objective was to obtain a type of concrete with comparable performance to ordinary concrete, but adapted to the new requirements of cost, energy efficiency, and sustainability. The tests performed included compressive strength at 28 days and 3 months, flexural tensile strength, splitting tensile strength, and the resistance to the action of repeated freeze–thaw cycles and to the action of hydrochloric acid.

## 2. Materials and Methods

### 2.1. Materials

The vegetal materials used in this research were: ash from corn cobs of quality A and quality B, and ash from sunflower stalks of quality A and quality B. The developed and analyzed compositions were as follows:

- A reference composition of micro concrete, RC, with cement, sand and river gravel aggregates up to 8 mm in diameter, with a water/cement ratio of 0.5;
- Eight concrete compositions with vegetal ash developed on the basis of RC:
  - With corn cob ash of A quality, as 2.5% and 5% replacement of cement volume (CCA1A, CCA2A),
  - With corn cob ash of B quality, as 2.5% and 5% replacement of cement volume (CCA1B, CCA2B),
  - With sunflower stalk ash of A quality, as 2.5% and 5% replacement of cement volume (SSA1A, SSA2A),
  - With sunflower stalks ash of B quality, as 2.5% and 5% replacement of cement volume (SSA1B, SSA2B).

The reference composition, RC, of 30/37 strength class, was realized for river gravel (sort 4–8 mm), sand (sort 0–4mm), cement Portland CEM II/A-LL42.5R type (notations according to EN 197-1:2011) [30] (HeidelbergCement Romania, Bucharest, Romania), superplasticizer additive based on polycarboxylateter (2% of binder), and water, according to NE 012/1-2007 [31].

### 2.2. Methods

#### 2.2.1. Vegetal Ash Preparation

Sunflower stalks (SS) were broken manually into pieces of around 0.5 m in length, and corn cobs (CC) were mechanically shredded with a mill for grinding animal feed in granules smaller than 6 mm in diameter. SS and CC were free burned in a brick kiln, after they were left to dry in outside natural conditions of the environment. The burning temperature was not controlled; it was measured, and it achieved around 700 °C in the case of SS case and 570 °C in the case of CC.

The raw ash obtained after free burning was sifted for 5 min through sieves of 20 mm, 10 mm, 2 mm, and 300 µm, using an automatic sieving equipment (Endocotts Powermatic Test Sieve Shaker). The 300 µm sieve was chosen as Bahuradeen et al., 2015 [10], stated that the pozzolanic properties of ash can be improved if the particles are smaller than this dimension.

**Figure 1:**
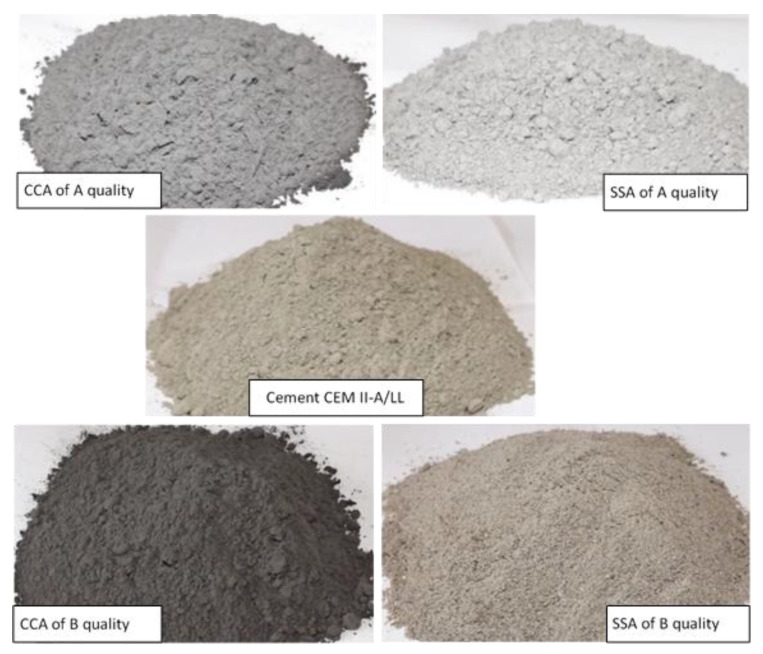
The SSA of A and B quality and CCA of A and B quality, compared with the cement aspect.

The sieved ash was then ground for 120 min in a ball dust crusher to obtain even smaller dimensions of the ash particles. The ground ash (Figure 1) was noted as A quality ash and it was used as cement partial replacement in CCA1A, CCA2A, SSA1A, and SSA2A concrete mixes.

The vegetal material that did not passed through the 300 µm sieve was subjected to supplementary thermal treatment at around 550 °C for 120 min. After this treatment, the vegetal material was sieved again through 20 mm, 10 mm, 2 mm, and 300 µm sieves for 5 min, with the same sieving equipment, and then ground for 120 min in the ball dust crusher. This ground ash (Figure 1) was noted as B quality ash and it was used as cement partial replacement in CCA1B, CCA2B, SSA1B, and SSA2B concrete mixes.

The ashes’ bulk density and specific gravity are presented in Table 1.

#### 2.2.2. Sunflower Stalk Ash Analysis

CCA and SSA were analyzed using Scanning Electron Microscopy (SEM, Vega Tescan LMH II, SE detector, 30 kV, Tescan Orsay Holding, Brno—Kohoutovice, Czech Republic) coupled with Energy Dispersive X-Ray Spectrometer detector (EDS, Bruker XFlash 6I30, Automatic mode, Bruker, Billerica, MA, USA) from composition and aspect point of view.2.

#### 2.2.3. Composite Mixes Preparation

In this study, concrete with 2.5 vol.% and 5.0 vol.% corn cob ash and sunflower stalk ash, of two qualities each, as a cement substitute were developed. The 9 mixes were prepared according to NE 012/1-2007 [31]. A portable electric concrete mixer was used for fresh mixes preparation. The concrete specimens were unmolded after 24 h from pouring in molds, and cured in ambient conditions for 28 days. The ambient conditions were: temperature of 20 ± 3 °C, and relative humidity of 55 ± 10%.

#### 2.2.4. Concrete Specimens Properties

##### Composition Analysis

The chemical elements and aspect of the developed concretes were analyzed through Scanning Electron Microscopy (SEM, VegaTescan LMH II, SE detector, 30 kV) coupled with Energy Dispersive X-Ray Spectrometer detector (EDS, Bruker XFlash 6I30, Automatic mode). A low vacuum mode was necessary to analyze the samples due to the fact that all samples gassed quite hard. Cubes samples (1 cm^3^ volume) were mechanically cut and used for analyses.

##### Mechanical and Durability Properties Analysis

In Table 2, the essential elements for the applied methods in performing tests are presented regarding the compressive strength, flexural tensile strength, splitting tensile strength, resistance to repeated freeze–thaw cycles, and resistance to hydrochloric acid action.

The specimens were prepared by casting in metal molds: cylinders with 100 mm diameter and 200 mm length, prisms of 100 × 100 × 550 mm, cubes with sides of 100 mm and 50 mm. The tests were performed at 28 days. In all tests, 3 specimens were used for each mixture and each test, according to the standards mentioned in Table 2, and the average value was calculated. Only for the freeze–thaw resistance test were six specimens used for each mix, as three specimens were the control samples, not subjected to the freeze–thaw cycles, and three specimens were subjected to the action of the freeze–thaw process.

##### Resistance to Repeated Freeze–Thaw Cycles

For the freeze–thaw test, cube specimens with sides of 100 mm were used, according to the SR 3518:2009 [35] standard. Before the start of the 50 freeze–thaw cycles, 6 specimens for each composition were immersed in water. The water had a temperature of 20 ± 5 °C. The immersion was prepared gradually, the water level being raised every 24 h, first up to ¼ of their height, then up to ½, then up to ¾, and finally to the total immersion. After the water immersion, three of the six specimens were kept in a water bath, whereas the other three were tested in 50 freeze–thaw cycles. During the freeze–thaw cycle of 8 h, for the first 4 h, the specimens were frozen in a cold room at the temperature of −17 ± 2 °C, and then for next 4 h, they were introduced in water with a temperature of 20 ± 5 °C.

After 50 cycles, the specimens subjected to freeze–thaw and those kept only in water were tested for compressive strength, in accordance with EN 12390-3:2019 [32] stipulations. The evaluation was conducted through the difference between the average compressive strength of the three specimens kept in water and the average compressive strength of the three specimens tested in 50 freeze–thaw cycles.

##### Resistance to Chemical Attack of Hydrochloric Acid (HCl)

**Figure 2:**
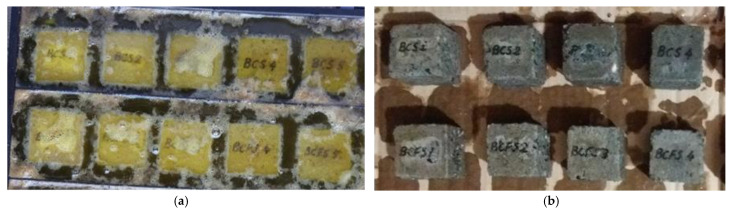
Resistance to the action of hydrochloric acid: (**a**) Specimens immersed in 18% HCl solution; (**b**) Specimens’ aspect after 10 days of immersion in 18% HCl solution.

Three cubes with side of 50 mm were used for each mix in performing the test for resistance to chemical attack of hydrochloric acid. The cubes were first dried in an oven at 90 °C until constant mass was reached, and then they were weighed. Afterwards, they were introduced in a HCl solution of 18% concentration (Figure 2a) for 10 days. This concentration level has been chosen in order to perform the test in a shorten period and to subject the mixes to an aggressive action of this acid. After a period of 10 days, the cubes were washed with clean water and brushed for the removal all loose particles (Figure 2b) and dried out in the oven at 90 °C till the constant mass was attained. Finally, the specimens were weighed. The resistance evaluation was performed by determining the average relative mass loss.

## 3. Results and Discussions

### 3.1. Vegetal Ashes and Cement

EDS analysis led to the quantitative elementary chemical composition of CCA and SSA presented in Table 3. As it can be seen in the cement composition, Si, Al, Ca and Fe are the predominant chemical elements. Compared with cement, if we look at the composition of the vegetal ashes, it can be observed that Si lacks the B quality of SSA, Al and Fe are present only in the B quality of CCA, and Ca content is a little smaller in the SSA at A and B quality and in a much smaller rate in the CCA at A and B quality. If we look to the other two elements present in cement, namely K and S, it can be observed that the K content is much higher (around 20–23 times), whereas the S content is quite similar or a little smaller in CCA and it lacks in SSA. In contrast to the cement, in both vegetal ashes supplementary elements appear, namely small rates of C, Mg, and Cl, and in CCA, S and P.

In a comparative analysis between CCA and SSA compositions, the following observations can be drawn:

- Si content in CCA is quite significant compared with SSA, being 10 times higher for A quality, whereas it is lacking in SSA at B quality;
- Only CCA at B quality has a small rate of Al and Fe;
- Ca content is around six times higher in SSA than in CCA;
- K quantities are very high in all ashes, around 21–23% in CCA and around 23–25% in SSA; the B quality of SSA has the bigger K content;
- S and P are present only in CCA;
- C content is much higher in CCA than in SSA, with 1.8 times higher for A-quality ash, and with 2.5 times higher for B quality;
- Mg content is almost double in SSA than in CCA, whereas the Cl rates does not differ significantly between them.

**Figure 3:**
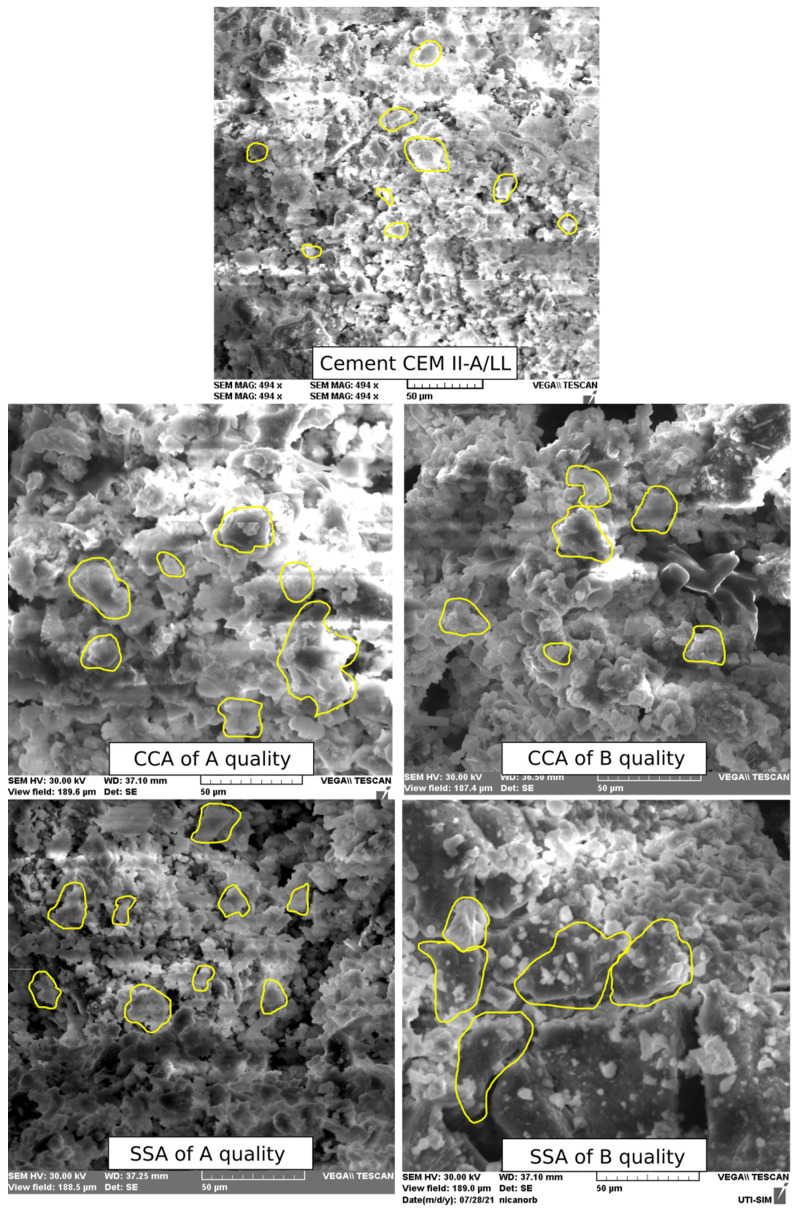
SEM images of CCA, SSA and cement at the magnification rate of 50 µm.

Scanning electron microscopy (SEM) images of A- and B-quality ashes of corn cobs and sunflower stalks are presented in Figure 3, at the 50 µm scale. SSA and CCA particles are bigger and less compact than the cement ones and present an agglomerated aspect (in Figure 3, some examples are marked). The aspect of SSA at B quality is more compacted than that of SSA at A quality. In the case of CCA, the aspect of the two variants has no big differences. The agglomeration of particles can be due to the high content of K, and it is in agreement with the results observed by Shakouri et al., 2020 [28], and Kamau et al., 2016 [5]. According to Shakouri et al., 2020 [28], the K content depends on the plant species and on the fertilizers used in crops.

**Figure 4:**
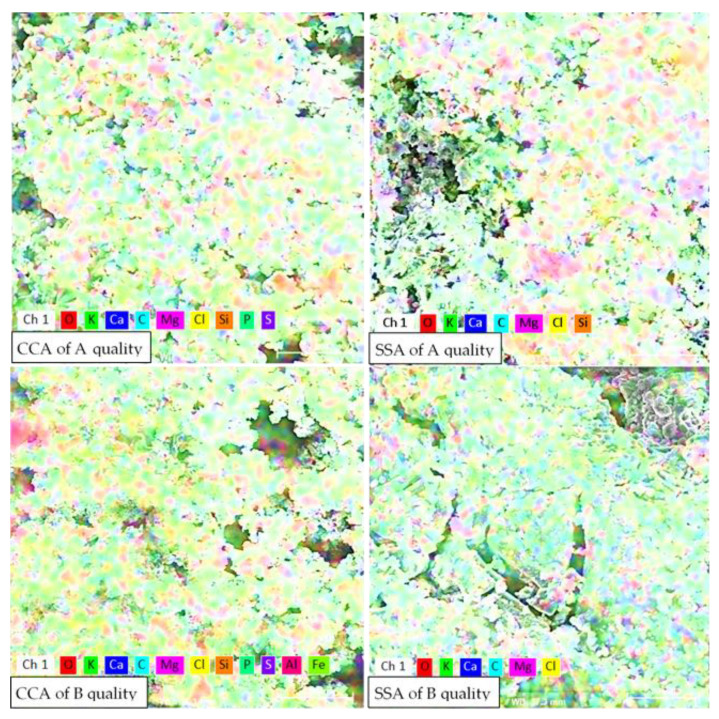
The distribution of the main identified elements in CCA and SSA, identified by EDS analysis, at the magnification rate of 80 µm.

In Figure 4 are presented the distributions of the main identified elements through EDS analysis from Table 3, on a 0.01 mm^2^ area, highlighting the main components of the A- and B-quality ashes of corn cobs and sunflower stalks and their distribution on the conglomerate. Beside the general oxide mass, a few small compounds based on Si or Cl can be observed.

### 3.2. Composite Specimens Properties

#### 3.2.1. Chemical Composition Analysis

In Table 4, the chemical composition of the studied concretes, according to EDS analysis, is presented from the mass and atomic percentage points of view.

The silicon content can be observed to be increased in almost all concretes with vegetal ashes than in the RC; only in SSA2B is it very insignificantly decreased. The carbon level is significantly decreased (up to 50%) in all mixes with vegetal ashes, except for SSA1A and SSA2B, which have very close values to RCs. The Calcium content is quite similar in CCA1A and CCA1B to that in RCs, being around 2–3% higher in CCA2A, CCA2B, and SSA1A, being almost 3% smaller in SSA2A, and it lacks in SSA1B. Aluminum levels are very close to that of RC in almost all mixes with vegetal ashes, except SSA2A and SSA1B, where they are almost four times higher. Regarding the Iron, there are no significant differences between the mixes. SSA2A contains a very small rate of Sodium, and in CCA2A, CCA2B, SSA2A, SSA1B, and SSA2B there exists small amounts of Potassium that are directly related to the strength performances of the concrete, even if its rate is very small [36].

**Figure 5:**
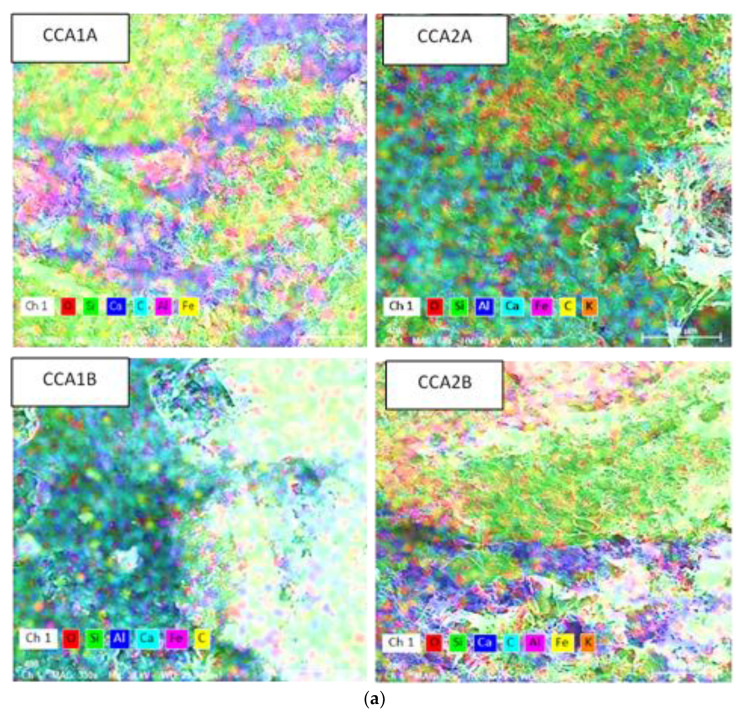

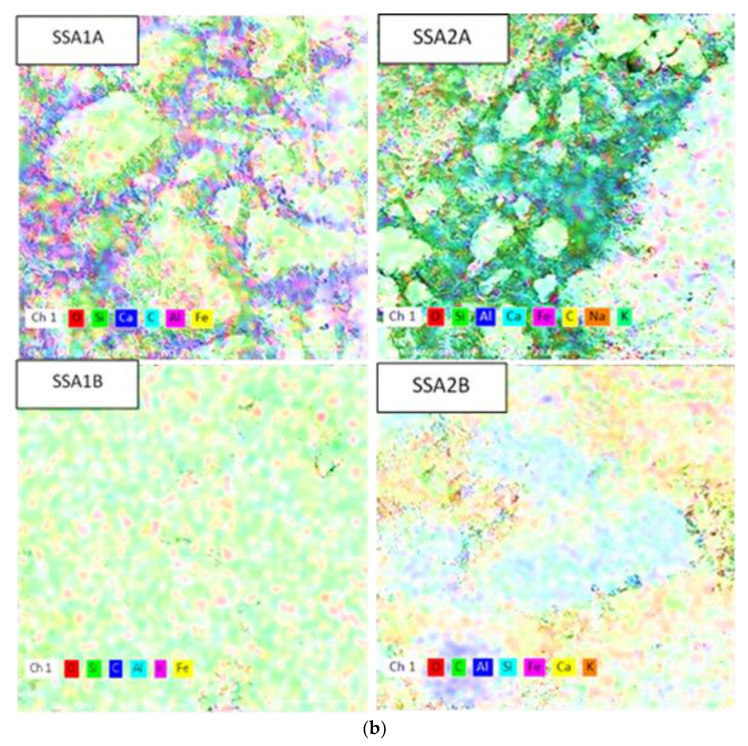
The distribution of the main identified elements in the studied concretes, at the magnification rate of 400 µm: (**a**) CCA concretes, (**b**) SSA concretes.

In Figure 5, the distribution of the main identified elements in the studied concretes is presented at a magnification rate of 400 µm. In SSA1A and CCA1A, the O, Si, Ca, C, Al, and Fe elements are visible, and the greener zones containing more Si (from the aggregates composition), and the bluer ones (given by the Ca element color from the legend of each image) are correspondent to calcium-silicate-hydrate (CSH) resulting from the pozzolanic reaction within the matrix. The C content contributes to the decreasing strength of the concrete. In SSA2A and CCA2A, the distribution of O, Si, Al, Ca, Fe, C, and K elements can be seen, according to the attached legend of attributed colors to each element, the bluer zones corresponding to the CSH content. The difference between those two mixes is the Na element present in SSA2A. In SSA1B, the Si, Al, and Fe content is more pronounced than the other elements, O, C, and K. In SSA2B, Al, Si and Ca are predominant. Additionally, the K element is quite visible. In CCA1A and CCA2B, the CSH zones are visible in the left half and bottom half of the images, respectively.

#### 3.2.2. Compressive Strength

**Figure 6:**
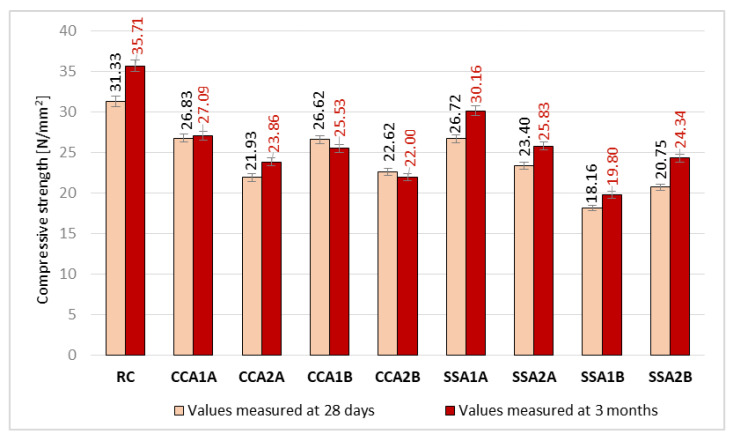
Compressive strength values at 28 days and 3 months age (N/mm^2^).

The compressive strength determined at 28 days is presented in Figure 6. As it can be seen in the graph, CCA1A, CCA1B, and SSA1A registered similar values for compressive strength, being around 14.7 ± 0.3% smaller than RC. Therefore, in the case of CCA, the qualities of the two types of ash led to almost the same result, and between CCA and SSA at A quality there are no big differences. CCA1A and CCA2A registered the best results between the ash mixes due to the Si content. SSA1A good result can be attributed to the smaller content of C in SSA than in CCA. Instead, B quality of SSA determined the biggest decrease in compressive strength than RC, with 42% in the case of concrete compositions with 2.5% ash. From the mixes with 5% vegetal ash, the best result was registered by SSA2A, followed by CCA2B and CCA2A, with 25.30%, 27.80%, and 30.00% smaller values than RC.

In conclusion, regarding the compressive strength measured at 28 days, for the 2.5% replacement rate of cement, the best result was given by the A quality of CCA, and for the 5% replacement rate, by the A quality of SSA.

Regarding the compressive strength evolution from 28 days to 3 months, according to Figure 6, it can be observed that the biggest increase was achieved by SSA2B, namely 14.74%, with around 12.5% bigger than that of RCs. SSA1A also had a good evolution, with a 11.40% increase, but with around 1% under the RCs. SSA2A, SSA1B, and CCA2A also registered good results, scoring 9.40%, 8.30%, and 8.10% more than the initial compressive strength. CCA at B quality instead determined a decrease in the time of this parameter, with around 3.5%.

If the value obtained at 28 days and evolution in time are considered together, the best result was obtained by SSA1A, which had a decrease in compressive strength of 14.70% than RC and a strength gain in time very close to that of RC’s, being only 0.9% smaller.

#### 3.2.3. Flexural Tensile Strength

**Figure 7:**
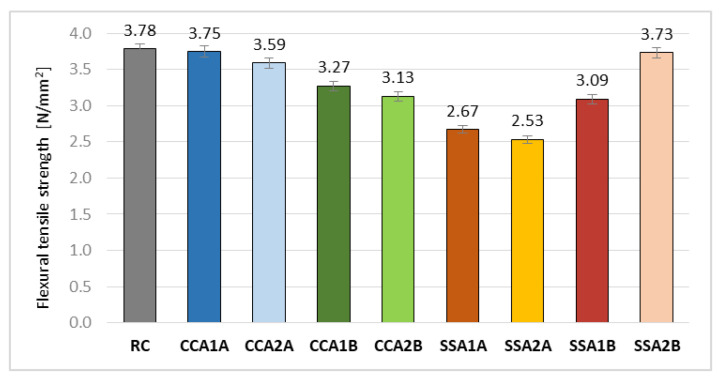
Flexural tensile strength values [N/mm^2^].

The vegetal ash used in the concrete composition led to a decrease in the flexural tensile strength (Figure 7). CCA1A and SSA2B registered the best results regarding this parameter, being only 0.95% and 1.30% smaller than RC. This can be attributed to the Si presence in CCA, and the higher content of Ca plus a much smaller C content in SSA, respectively. In CCA group, the ash at B quality led to smaller values than that at A quality, at around 12%. The SSA group registered more inferior results than the CCA one, especially in the case of using A-quality ash. SSA1A and SSA2A achieved a smaller flexural tensile strength with 29.5% and 33%, respectively, than RC. SSA at B quality obtained better results for this parameter than that of A quality, especially in the case of the 5% replacement rate.

In conclusion, the A-quality ash from corn cobs led to the best values among the studied mixes with vegetal ash, regarding the flexural tensile strength.

#### 3.2.4. Splitting Tensile Strength

**Figure 8:**
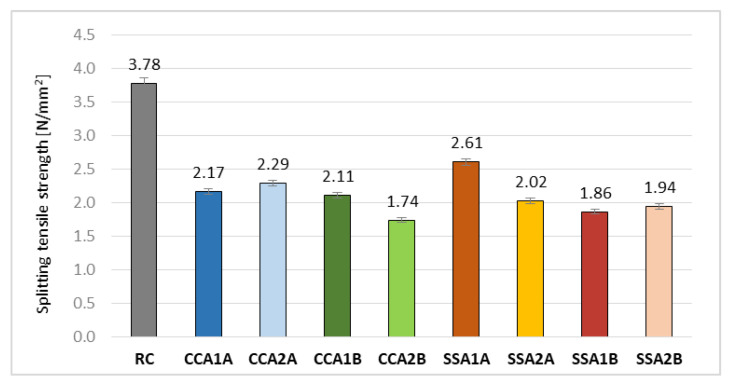
Splitting tensile strength values [N/mm^2^].

The corn cob and sunflower stalk ash had significant negative effects on the splitting tensile strength of the RC (Figure 8). The best result among the mixes with vegetal ash was obtained by SSA1A, being around 31% smaller than the RC’s results, followed by CCA2A, with a decrease of 39.4%. The best result for SSA1A can be attributed to a combination of elements: the higher Ca content in SSA at A quality for all ashes, its smallest C content, and Si presence. CCA1A, CCA1B, and SSA2A registered smaller values, with 43%, 44%, and 46.6%, respectively, than the reference. CCA2B obtained the biggest decrease, at around 54%.

#### 3.2.5. Resistance to Repeated Freeze–Thaw Cycles

**Figure 9:**
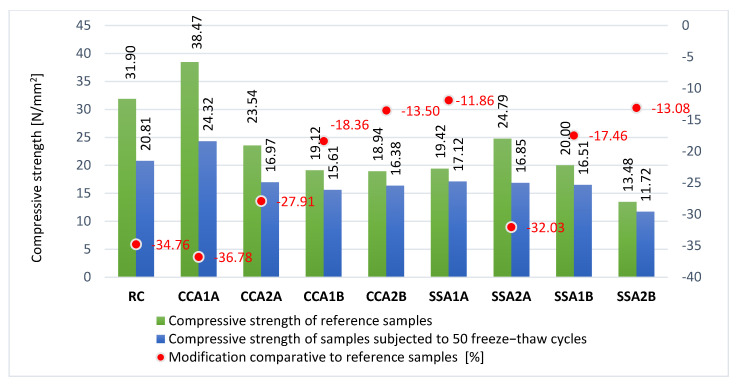
Compressive strength variation after 50 freeze–thaw cycles [%].

After 50 freeze–thaw cycles, RC registered a 34.80% decrease in compressive strength (Figure 9). A 2.5% replacement rate of cement with SSA of A quality led to a significant improvement of this parameter, this composition obtaining a 11.90% decrease only compared with control samples. The SSA1A performance can be attributed to a combination of chemical elements of SSA of A quality than the other ashes, namely the highest Ca rate, the smallest C content, and Si presence. The 5% B-quality ash of CCA and SSA also had positive effects, these mixes registering a 13.3 ± 0.2% decrease. CCA1B also obtained better freeze–thaw resistance than RC, but in a smaller rate than CCA2B and SSA2B, respectively; therefore, the conclusion can be drawn that a higher replacement rate of cement with B-quality ash for both plant cases led to improved freeze–thaw resistance. The good results obtained by CCA1B and CCA2B can be due to the Al and Fe content in the CCA at B quality. Regarding the A-quality ash, a higher replacement rate in the case of CCA led to an improvement of about 9%, but in the case of SSA led to a significant decrease from 11.9% up to 32%. Only CCA1A registered a smaller resistance, at around 2% (Figure 9).

#### 3.2.6. Resistance to HCl Chemical Attack

**Figure 10:**
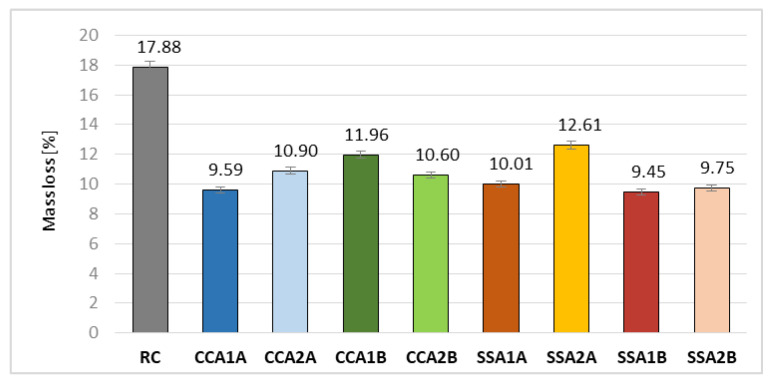
Mass loss due in case of chemical attack of 18% HCl solution [%].

All compositions with vegetal ash registered an improved resistance to chemical attack of 18% HCl solution, with more than 29.50% (Figure 10). The best results were obtained by SSA1B, CCA1A, SSA2B, and SSA1A, registering higher values with 45.5 ± 1.5% than RC. The good results obtained by CCA1A can be attributed to the highest Si content of CCA at A quality, whereas those of SSA1B and SSA2B to the combination of high content of Ca and small C content of the SSA at B quality. CCA2A and CCA2B registered a 40 ± 1% improvement than RC, whereas the results for CCA1B and SSA2B were 33% and 29.50%, respectively.

In CCA group, A quality ash led to better results than the B one, and an increased replacement rate of this ash type led also to improved resistance. In SSA group, B quality ash registered better values for resistance to the action of 18% HCl solution than the A one, but the improvement decreased as the replacement rate increased (Figure 10).

In conclusion, 2.5% of A quality of CCA and B quality of SSA led to the best values of resistance to 18% HCl solution (Figure 10).

## 4. Conclusions

This study aimed to analyze the effects of two different qualities of ashes obtained by corn cobs and sunflower stalks free burning, on some properties of a micro concrete, by applying 2.5 vol.% and 5 vol.% replacement of the cement. The analyzed properties were compressive strength, flexural tensile strength, splitting tensile strength, freeze–thaw resistance, and resistance to chemical attack of hydrochloric acid solution:

- The best results for compressive strength between all mixes with vegetal ash were obtained by CCA1A, SSA1A, and CCA1B, with very close values between them. Therefore, the 2.5% of CCA, no matter its quality, and 2.5% of SSA of A quality led to similar compressive strength values. Between these three variants of mixes with vegetal ashes, only SSA1A obtained a very good gain in compressive strength during a period of 3 months, very close to that of RC.
- Regarding flexural tensile strength, CCA1A and SSA2B registered the best results, with a more insignificant decrease than RC of only about 1%.
- CCA and SSA, no matter of the quality used, determined decreases in splitting tensile strength of RC, between 31% and 54%. The smallest decreases were registered by SSA1A and CCA2A, with 31% and 39%, respectively, smaller than the reference.
- All vegetal ashes used in this study led to very significant improvement of resistance to freeze–thaw and to the chemical attack of HCl. Regarding resistance to repeated freeze–thaw cycles, the biggest improvements were obtained by SSA1A, followed very closely by SSA2B and CCA2B, with 23–23% smaller losses in compressive strength than RC. Regarding resistance to chemical attack of 18% HCl solution, all studied mixes with vegetal ash obtained improvements of this parameter, between 30% and 47% more than the reference.
- CCA1A led to the smallest decreases in compressive strength at 28 days and flexural tensile strength, and it registered one of the biggest values regarding resistance to HCl action.
- SSA1A was the optimum mix regarding compressive strength at 28 days and 3 months, splitting tensile strength and freeze–thaw resistance, and it obtained a very significant improvement of resistance to HCl, at 44%.
- SSA1B obtained the biggest improvement of the resistance from the HCl action point of view, and one of the best results from the freeze–thaw resistance point of view.
- CCA2B and SSA2B obtained very significant increases as resistance to freeze–thaw cycles and to HCl action.
- SSA2B registered the biggest compressive strength gain in time, bigger than RC, and one of the smallest decreases in flexural tensile strength.

Most concrete and reinforced concrete applications are based on the idea of improving the strength properties. However, the role of using corn cob and sunflower stalk ash as partial substitutes for cement is not to improve the mechanical strength, but to improve other properties of concrete, such as resistance to repeated freeze–thaw cycles or resistance to various chemical agents, properties that make them more economical in terms of production costs and more environmentally friendly by reducing the amount of energy incorporated in their production. If engineers stop thinking about strength, it will not be difficult to find many other areas of application for these new materials, as well as improving them at the same time.

Regarding applications for concrete with the studied vegetal ashes, the chloride content of CCA and SSA limits their use to unreinforced concrete applications or to applications with non-corrosive reinforcement or non-structural ones.

As future directions to be researched, the effects of bigger rates of CCA and SSA as cement replacement or as additive material on the freeze–thaw resistance can be studied; in general, in this study, bigger improvements were observed as the vegetal ash content increased.

## Figures and Tables

**Table 1:** The bulk density and specific gravity of CCA and SSA.

Ash Type	Bulk Density (g/L)	Specific Gravity (N/m^3^)
CCA of A quality	725	2.102
CCA of B quality	699	2.027
SSA of A quality	805	2.334
SSA of B quality	799	2.317

**Table 2:** Mechanical and durability test details.

Test	Specimens’ Type	Specimens’ Dimensions	Specimens Number Tested for Each Mix	Standard Applied
Compressive strength	Cylinder	100 mm diameter 200 mm length	3	EN 12390-3: 2019 [32]
Flexural tensile strength	Prism	100 × 100 mm^2^ transversal section 550 mm length	3	EN 12390-5: 2019 [33]
Splitting tensile strength	Cylinder	100 mm diameter 200 mm length	3	EN 12390-6: 2010 [34]
Resistance to freeze–thaw	Cube	sides of 100 mm	6	SR 3518: 2009 [35] EN 12390-3: 2019 [32]
Resistance to hydrochloric acid action	Cube	sides of 50 mm	3	Figure 2

**Table 3:** CCA, SSA and cement elementary chemical composition, quantitative identification.

Element	CCA				SSA				Cement	
	A		B		A		B			
	Mass Norm. (%)	Atom (%)	Mass Norm. (%)	Atom (%)	Mass Norm. (%)	Atom (%)	Mass Norm. (%)	Atom (%)	Mass Norm. (%)	Atom (%)
Oxygen (O)	47.58	56.28	39.56	45.81	50.81	63.76	52.85	65.29	47.65	64.17
Silicon (Si)	7.14	4.81	5.42	3.58	0.68	0.49	-	-	20.97	16.09
Aluminum (Al)	-	-	1.76	1.21	-	-	-	-	12.89	10.29
Calcium (Ca)	1.63	0.77	1.41	0.65	10.18	5.10	8.94	4.41	12.12	6.51
Iron (Fe)	-	-	1.14	0.38	-	-	-	-	4.25	1.64
Potassium (K)	21.22	10.27	22.75	10.78	22.85	11.73	25.00	12.64	1.07	0.59
Sulfur (S)	1.18	0.70	0.69	0.40	-	-	-	-	1.05	0.70
Carbon (C)	14.62	23.64	22.13	34.13	7.91	13.23	8.97	14.77	-	-
Magnesium (Mg)	1.83	1.43	1.13	0.86	5.41	4.47	2.06	1.68	-	-
Chlorine (Cl)	3.03	1.62	2.50	1.31	2.16	1.22	2.18	1.21	-	-
Phosphorus (P)	1.77	1.08	1.51	0.90	-	-	-	-	-	-
SUM	100	100	100	100	100	100	100	100	100	100

**Table 4:** Chemical composition of the studied concretes, according to EDS analysis.

Element	RC		CCA1A		CCA2A		CCA1B		CCA2B		SSA1A		SSA2A		SSA1B		SSA2B	
	Mass [%]	Atom [%]	Mass [%]	Atom [%]	Mass [%]	Atom [%]	Mass [%]	Atom [%]	Mass [%]	Atom [%]	Mass [%]	Atom [%]	Mass [%]	Atom [%]	Mass [%]	Atom [%]	Mass [%]	Atom [%]
Oxygen	53.96	61.22	53.33	64.29	53.62	64.10	53.01	63.51	53.69	64.01	52.34	60.75	51.54	62.22	51.92	62.15	52.07	59.86
Silicon	20.69	13.38	26.63	18.29	20.80	14.17	25.79	17.60	21.24	14.42	20.94	13.84	24.86	17.10	35.58	24.26	20.15	13.20
Carbon	13.02	19.68	6.74	10.82	8.27	13.16	7.71	12.30	8.40	13.33	11.80	18.24	7.22	11.61	6.09	9.71	13.05	19.98
Calcium	10.39	4.71	10.26	4.94	13.75	6.56	10.25	4.90	13.34	6.35	12.13	5.62	7.74	3.73	-	-	11.26	5.17
Aluminum	1.13	0.76	1.67	1.20	1.89	1.34	1.58	1.12	1.82	1.29	1.75	1.20	4.04	2.89	4.04	2.87	1.49	1.02
Iron	0.81	0.26	1.37	0.47	1.03	0.35	1.66	0.57	0.91	0.31	1.04	0.35	2.02	0.70	1.01	0.35	1.13	0.37
Sodium	-	-	-	-	-	-	-	-	-	-	-	-	1.39	1.17	-	-	-	-
Potassium	-	-	-	-	0.63	0.31	-	-	0.61	0.30	-	-	1.19	0.59	1.36	0.67	0.85	0.40
SUM	100	100	100	100	100	100	100	100	100	100	100	100	100	100	100	100	100	100

## Data Availability

Data is contained within the article.

## References

[1] Rui Guo R., Wang J., Bing L., Dan Tong D., Ciais P., Davis S.J., Andrew R.M., Xi F., Liu Z. (2020). Global CO_2_ uptake of cement in 1930–2019. Earth Syst. Sci. Data.

[2] Hasanbeigi A. (2021). Global Cement Industry’s GHG Emissions. https://www.globalefficiencyintel.com/new-blog/2021/global-cement-industry-ghg-emissions.

[3] Garside M. (2021). Major Countries in Worldwide Cement Production 2010–2020. https://www.statista.com/statistics/267364/world-cement-production-by-country.

[4] Gambhir (2009). Concrete Technology 4e.

[5] Kamau J., Ahmed A., Hirst P., Kangwa J. (2016). Suitability of Corncob Ash as a supplementary Cementitious Material. Int. J. Mater. Sci. Eng..

[6] Bie R.S., Song X.F., Liu Q.Q., Ji X.Y., Chen P. (2015). Studies on effects of burning conditions and rice husk ash (RHA) blending amount on the mechanical behavior of cement. Cem. Concr. Compos..

[7] Antiohos S.K., Tapali J.G., Zervaki M., Sousa-Coutinho J., Tsimas S., Papadakis V.G. (2013). Low embodied energy cement containing untreated RHA: A strength development and durability study. Constr. Build. Mat..

[8] Chindaprasirt P., Rukzon S., Sirivivatnanon V. (2008). Resistance to Chloride Penetration of Blended Portland cement Mortar Containing Palm Oil Fuel Ash, Rice Husk Ash and Fly Ash. Constr. Build. Mat..

[9] Amin M.N., Khan K., Aslam F., Shah M.I., Javed M.F., Musarat M.A., Usanova K. (2021). Multigene Expression Programming Based Forecasting the Hardened Properties of Sustainable Bagasse Ash Concrete. Materials.

[10] Bahurudeen A., Kanraj D., Gokul Dev V., Santhanam M. (2015). Performance evaluation of sugarcane bagasse ash blended cement in concrete. Cem. Concr. Compos..

[11] Bheel N., Adesina A. (2021). Influence of Binary Blend of Corn Cob Ash and Glass Powder as Partial Replacement of Cement in Concrete. Silicon.

[12] Adesanya D.A., Raheem A.A. (2009). Development of corn cob ash blended cement. Constr. Build. Mater..

[13] Memon S.A., Javed U., Haris M., Khushnood R.A., Kim J. (2021). Incorporation of Wheat Straw Ash as Partial Sand Replacement for Production of Eco-Friendly Concrete. Materials.

[14] Aksoğan O., Binici H., Ortlek E. (2016). Durability of concrete made by partial replacement of fine aggregate by colemanite and barite and cement by ashes of corn stalk, wheat straw and sunflower stalk ashes. Constr. Build. Mater..

[15] Martirena F., Monzó J. (2018). Vegetable ashes as supplementary cementitious materials. Cem. Concr. Res..

[16] Shafigh P., Mahmud H.B., Jumaat M.Z., Zargar M. (2014). Agricultural wastes as aggregate in concrete mixtures—A review. Constr. Build. Mater..

[17] OECD (Organisation for Economic Cooperation and Development) Crop Production (Indicator). https://www.oecd-ilibrary.org/agriculture-and-food/crop-production/indicator/english_49a4e677-en.

[18] USDA (United States Department of Agriculture) World Agricultural Production. Circular Series WAP 10-21 2021, 38. https://apps.fas.usda.gov/psdonline/circulars/production.pdf.

[19] Leff B., Ramankutty N., Foley J.A. (2004). Geographic distribution of major crops across the world. Glob. Biogeochem. Cycles.

[20] Adesanya D. (1996). Evaluation of blended cement mortar, concrete and stabilized earth made from ordinary Portland cement and corn cob ash. Constr. Build. Mater..

[21] Binici H., Yucegok F., Aksogan O., Kaplan H. (2008). Effect of corncob, wheat straw, and plane leaf ashes as mineral admixtures on concrete durability. J. Mater. Civ. Eng..

[22] Kamau J., Ahmed A., Hirst P., Kangwa J. (2017). Permeability of corncob ash, anthill soils and rice husk replaced concrete. Int. J. Sci. Environ. Technol..

[23] Memon S.A., Khan M.K. (2018). Ash blended cement composites: Eco-friendly and sustainable option for utilization of corncob ash. J. Clean. Prod..

[24] Oladipupo O.S., Olutoge F.A. (2012). Strength Properties of Corn Cob Ash Concrete. J. Emerg. Trends Eng. Appl. Sci..

[25] Mujedu K.A., Adebara S.A., Lamidi I.O. (2014). The Use of Corn Cob Ash and Saw Dust Ash as Cement. Int. J. Eng. Sci..

[26] Ahangba A., Michael T. (2016). Partial replacement of cement with corn cob ash. Int. J. Innov. Res. Multidiscip. Field.

[27] Owolabi T.A., Oladipo I.O., Popoola O.O. (2015). Effect of corncob ash as partial substitute for cement in concrete. N. Y. Sci. J..

[28] Shakouri M., Exstrom C.L., Ramanathan S., Suraneni P. (2020). Hydration, strength, and durability of cementitious materials incorporating untreated corn cob ash. Constr. Build. Mater..

[29] Darweesh H.H.M. (2020). Influence of sun flower stalk ash (SFSA) on the behavior of Portland cement pastes. Results Eng..

[30] EN 197-1:2011 (2011). Cement. Composition, Specifications and Conformity Criteria for Common Cements.

[31] NE 012/1-2007 (2007). Normativ Pentru Producerea Betonului Și Executarea Lucrarilor Din. Beton, Beton Armat Și Beton Precomprimat—Partea 1: Producerea Betonului (Regulations for the Production of Concrete and the Execution of Concrete, Reinforced Concrete and Prestressed Concrete).

[32] EN 12390-3:2019 (2019). Testing Hardened Concrete Part. 3: Compressive Strength of Test. Specimens.

[33] EN 12390-5:2019 (2019). Testing Hardened Concrete Part. 3: Flexural Tensile Strength of Test. Specimens.

[34] EN 12390-6:2010 (2010). Testing Hardened Concrete; Part. 6: Split Tensile Strength of Test. Specimens.

[35] SR 3518:2009 (2009). Încercări Pe Betoane. Determinarea Rezistenței La Îngheț-Dezgheț Prin Masurarea Variației Rezistenței La Compresiune¸ Si/Sau Modulului de Elasticitate Dinamic Relativ (Tests Hard-Ened Concrete. Determination of Freeze-Thaw Resistance by Measuring the Variation of the Com-Pressive Strength and/or the Relatively Dynamic Modulus of Elasticity).

[36] Neville A.M. (2012). Properties of Concrete.

